# Donated Human Milk as a Determinant Factor for the Gut Bifidobacterial Ecology in Premature Babies

**DOI:** 10.3390/microorganisms8050760

**Published:** 2020-05-19

**Authors:** Silvia Arboleya, Silvia Saturio, Marta Suárez, Nuria Fernández, Leonardo Mancabelli, Clara G. de los Reyes-Gavilán, Marco Ventura, Gonzalo Solís, Miguel Gueimonde

**Affiliations:** 1 Department of Microbiology and Biochemistry of Dairy Products, Instituto de Productos Lácteos de Asturias (IPLA-CSIC), 33300 Villaviciosa, Spain; Silvia.Saturio@ipla.csic.es (S.S.); greyes_gavilan@ipla.csic.es (C.G.d.l.R.-G.); mgueimonde@ipla.csic.es (M.G.); 2 Diet, Human Microbiota and Health Group, Institute of Health Research of the Principality of Asturias (ISPA), 33011 Oviedo, Spain; nuriajmhd@gmail.com; 3 Department of Pediatrics Service, Central University Hospital of Asturias (HUCA-SESPA), 33011 Oviedo, Spain; msr1070@hotmail.com (M.S.); gsolis@telefonica.net (G.S.); 4 Laboratory of Probiogenomics, Department of Chemistry, Life Sciences and Environmental Sustainability, University of Parma, 43121 Parma, Italy; leonardo.mancabelli@genprobio.com (L.M.); marco.ventura@unipr.it (M.V.)

**Keywords:** bifidobacteria, ITS, intestinal microbiota, preterm, mother’s own milk, donated human milk, early life

## Abstract

Correct establishment of the gut microbiome is compromised in premature babies, with *Bifidobacterium* being one of the most affected genera. Prematurity often entails the inability to successfully breastfeed, therefore requiring the implementation of other feeding modes; breast milk expression from a donor mother is the recommended option when their own mother’s milk is not available. Some studies showed different gut microbial profiles in premature infants fed with breast milk and donor human milk, however, it is not known how this affects the species composition of the genus *Bifidobacterium*. The objective of this study was to assess the effect of donated human milk on shaping the gut bifidobacterial populations of premature babies during the first three months of life. We analyzed the gut bifidobacterial communities of 42 premature babies fed with human donor milk or own-mother milk by the 16S rRNA–23S rRNA internal transcriber spaces (ITS) region sequencing and q-PCR. Moreover, metabolic activity was assessed by gas chromatography. We observed a specific bifidobacterial profile based on feeding type, with higher bifidobacterial diversity in the human donor milk group. Differences in specific *Bifidobacterium* species composition may contribute to the development of specific new strategies or treatments aimed at mimicking the impact of own-mother milk feeding in neonatal units.

## 1. Introduction

In premature infants, the correct process of gut microbiota colonization is compromised due to several factors, such as their immature immune systems and gut mucosa, long stays in hospitals, various medications, particularly antibiotics, etc. [1]. These factors result in increased gut permeability, reducing the gut barrier and increasing the risk of infections and disease, in addition to other complications associated with decreasing gestational age [2]. Several studies showed how gut microbiota establishment in premature and extremely premature (or very low birth weight) babies is affected with respect to full-term babies, demonstrating lower diversity and increased levels of Enterobacteriaceae members and putative pathogens like *Klebsiella pneumoniae* and *Clostridioides difficile*. Delayed colonization by beneficial microbes such as *Bifidobacterium* during early life was also observed [3,4]. Bifidobacteria are some of the first colonizers of the gut microbiota and are dominant in fecal samples of healthy breastfed infants, associated with the consumption of human milk oligosaccharides (HMOs) [1,5]. Moreover, bifidobacteria are essential in maintaining gut homeostasis and exert an important role in human health maintenance from the early stages of life [6,7]. 

Premature infant care is in continuous progress; however, optimal feeding is still a challenge due to specific requirements and most extremely premature babies suffer postnatal growth retardation [8]. Breastfeeding and human milk are the gold standards for infant feeding and nutrition and are recommended from the first hour of life to the first six months exclusively, with complementary solid foods being introduced up to two years of age or beyond [9]. Breast milk contains all the compounds and energy necessary for the correct development and growth of infant during the early stages of life and is associated with the promotion of neurodevelopment, protective effects against necrotizing enterocolitis (NEC), and reductions in late onset sepsis and retinopathy severity in prematurity [8,10]. 

Breastfeeding remains the desired mode of infant feeding in difficult situations, such as in premature babies; however, prematurity often makes mothers of such babies unable to successfully breastfeed, requiring the implementation of alternative feeding modes. In this situation, the WHO recommends using expressed breast milk from donor mothers as a preference to formula milk in premature babies, including healthy but very low birth weight babies until 1 kg [11]. Donor human milk (DHM) is associated with lower incidence of severe gut disorder, NEC, and other infections during initial hospital stays after birth in comparison with formula feeding [8,11]. In hospitals where milk-banking facilities are available and in other external milk banks, DHM is subjected to the holder pasteurization (HoP) process, which involves raising the temperature of human milk to 62.5 °C for 30 minutes to ensure the inactivation of bacteria and viruses [12]. Thus, the HoP process entails modification of the raw maternal milk components such as bacterial and human cells, immunoglobulins, enzymes, proteins, cytokines, hormones, growth factors, and antioxidants [8], but some authors maintain that no modifications occur in the saccharide component [8,13]. Therefore, HoP is still a matter of debate. Human milk is not a sterile fluid, but its microbiota is eliminated by the HoP process. Premature babies fed with DHM were observed to have decreased gut microbial diversity and showed different gut microbial profiles with respect to those fed with their own mother’s milk (OMM) [14,15,16]. Whether the lack of beneficial milk bacteria or the modification of important components, such as HMOs, affects gut microbiota is still unknown. Previous studies showed alterations in the *Bifidobacterium* genus, however, how DHM affects the species composition of this important genus at the beginning of life is still unclear.

The aim of this study was to assess the effect of donated human milk on shaping the gut bifidobacterial populations in premature babies during the first three months of life.

## 2. Materials and Methods 

### 2.1. Volunteers and Collection of Fecal Samples 

We carried out a prospective and observational study including 42 Caucasian premature babies recruited at the Central University Hospital of Asturias (HUCA, Northern Spain). The study was approved by the Regional Ethical Committee of Asturias Public Health Service (SESPA) (27 February, 2018) and informed written consent was obtained from each infant’s parents. All premature babies were exclusively fed human milk for at least the first ten days of life, either with their OMM or donated milk. At 30 days of life, half of the babies were receiving formula, with only three babies remaining in the OMM regimen at 90 days (Figure S1). None of the babies were engaged in mixed feeding and none received probiotics, prebiotics, or other treatments. Moreover, none of the infants presented with NEC, culture-positive early-onset infection, major malformations, or surgery of the intestinal tract, as per our exclusion criteria. All infants were discharged from the hospital after 47 ± 28 (mean ± SD) days of life.

Expressed donated human milk was obtained from term and preterm mothers (none from this study) and frozen immediately. Then, the milk was thawed, subjected to a HoP process and frozen until use, in accordance with the procedures at the HUCA Breast Milk Bank.

Fecal samples were collected at 2 (between 24 and 48 h after birth), 10, 30, and 90 days of life at the hospital. When infants were not at the Neonatal Care Unit (NCU), samples were collected after a scheduled appointment at the hospital by pediatricians. Samples were gathered in a sterile container from diapers, immediately frozen at −20 °C, and transported to the laboratory.

### 2.2. Analyses of Intestinal Bifidobacterial Species

#### 2.2.1. Analysis of Fecal Bifidobacterial Populations by ITS Region Profiling

Fecal samples were homogenized (1:10 w/v) in sterile phosphate-buffered saline (PBS) solution and centrifuged for 15 min at full speed. Cell pellets and supernatants were kept separately at −20 °C for further analysis. DNA samples were isolated from fecal pellets following Qiagen manufacturer’s instructions (QIAmp DNA stool kit, Qiagen GmbH, Hilden, Germany). Extracted DNA samples were kept at −20 °C until use for 16S rRNA–23S rRNA intergenic ribosomal transcriber spaces (ITS) and qPCR analyses.

Partial ITS regions were amplified from extracted DNA by PCR using specifically designed primers [17] and sequenced using the MiSeq (Illumina) platform at GenProbio srl (Italy). Following sequencing, the fastq files were processed using a custom script based on the QIIME2 software suite [18]. Paired-end reads were assembled to reconstruct the complete Probio-bif_Uni/Probio-bif_Rev amplicons. Quality control retained sequences with lengths between 100 and 400 bp and mean sequence quality scores of >20, while sequences with homopolymers > 7 bp in length and mismatched primers were removed. ITS Operational Taxonomic Units (OTUs) were defined at 100% sequence homology using DADA2 [19]. All reads were classified to the lowest possible taxonomic rank using QIIME2 [18,20] and a reference dataset, consisting of an updated version of the bifidobacterial ITS database [17]. The number of reads and relative abundances were determined for each bifidobacterial species in each analysed sample.

#### 2.2.2. Analysis of Fecal Bifidobacterial Populations by Specific Quantitative PCR

Absolute levels of the most relevant gut bifidobacterial species were determined by qPCR using the primers shown in Table 1. All reactions were carried out in a 7500 Fast Real Time PCR System (Applied Biosystems) using MicroAmp optical plates (Applied Biosystems, Foster City, CA). One microliter of fecal DNA, 0.2 µM of each primer and 2x SYBR Green PCR Master Mix (Applied Biosystems) were used in a final volume of 25 µL. Thermal cycling consisted of an initial cycle of 95 °C 10 min, followed by 40 cycles of 95 °C 15 s and 1 min at the appropriate primer-pair temperature (Table 1). The standard curves were made with pure cultures of each *Bifidobacterium* species grown in MRSC broth (deMann Rogosa and Sharp broth (BioKar Diagnostics, Beauvais, France) supplemented with 0.25% (w/v) L-cysteine (Sigma Chemical Co., St. Louis, MO, USA)) under anaerobic conditions. Samples were analyzed in duplicate in at least two independent PCR runs. 

### 2.3. Determination of SCFA Levels in Fecal Water

Short-chain fatty acids (SCFA) were analyzed by gas chromatography (GC). Cell-free supernatants from fecal homogenates were mixed with methanol, 20% (*v*/*v*) formic acid, and an internal standard solution (2-ethylbutyric) in a 38:46:8:8 (*v*/*v*) proportion. This mixture was used for SCFA quantification in a system composed of a 6890NGC injection module (Agilent Technologies Inc., Palo Alto, CA, USA) connected to a flame injection detector (FID) and a mass spectrometry (MS) 5973N detector (Agilent), as described elsewhere [24].

### 2.4. Statistical Analysis

ITS-sequencing results were analyzed using the Calypso software (version 8.84) [25] with total sum normalization (TSS) and cumulative-sum scaling (CSS) to account for the non-normal distribution of taxonomic count data [26]. Multivariate redundancy analysis (RDA) and discriminant analysis of principal components (DAPC) were conducted. Alpha diversity indexes (Chao1 and Shannon) were also calculated and analyzed by ANOVA using normalized data. In order to assess the differences in bifidobacterial gut relative abundance and further avoid potential issues with compositional data [27], the ALDEx2 function [28] was utilized using non-normalized data. LefSe test (linear discriminant analysis effect size) was used to detect species features between groups and the species core was calculated. Data from qPCR and SCFAs were analyzed using IBM SPSS Statistics (IBM Corp., Armonk NY, USA) software with the U-Mann–Whitney and Kruskal–Wallis tests. Perinatal factors were assessed using the U-Mann–Whitney and χ^2^ tests. Data were considered statistically significant at *p* < 0.05.

### 2.5. Nucleotide Sequence Accession Numbers

The raw sequences from the samples were deposited in the Nacional Center for Biotechnology Information Short Read Archive under the BioProject ID code PRJNA616077.

## 3. Results

### 3.1. Volunteer Characteristics

Forty-two Caucasian premature babies (24 males/18 females) were included in this study. Eighteen premature babies were delivered vaginally and 24 by Caesarean section (C-section). They were born at gestational ages between 24 and 34 weeks (mean 31 ± 2) and their birth weights were 1334.88 ± 338.64 g (mean ± SD). They were discharged after 47 ± 28 days (mean ± SD). Nine premature infants received antibiotics at birth and 39 of the premature babies’ mothers received intrapartum antibiotics. Twenty-eight infants were breastfed with OMM during the first 10 days of life, whereas 13 premature received DHM from the Breast Milk HUCA Bank at the hospital. At 30 days of life, five babies continued received DHM and 13 received OMM; the other babies received formula. At 90 days most of the premature babies were formula-fed, except three babies who were breastfeeding since birth (Table S1).

### 3.2. Impact of Donor Human Milk on Gut Bifidobacterial Composition

ITS bifidobacterial profiling analyses were carried out on 141 samples belonging to the 42 babies. Raw sequences were processed to classify reads into clusters of identical sequences (OTUs) and 31 bifidobacterial species were identified in the feces of the premature babies. In detail, we focused our interest on OTUs belonging to the 27 species most present in the babies (*B. adolescentis; B. animalis* spp. *animalis; B. animalis* spp. *lactis; Bifidobacterium anseris; Bifidobacterium asteroides; Bifidobacterium biavatii; B. bifidum; B. breve; B. catenulatum; Bifidobacterium choerinum; Bifidobacterium crudilactis; B. dentium; Bifidobacterium italicum; B. longum* spp. *infantis; B. longum* spp. *longum; B. longum* spp. *suis; Bifidobacterium magnum; Bifidobacterium mongoliense; Bifidobacterium parmae; Bifidobacterium pseudocatenulatum; Bifidobacterium pseudolongum* spp. *globosum; Bifidobacterium pseudolongum* spp. *pseudolongum; Bifidobacterium reuteri; Bifidobacterium thermacidophilum* spp. *porcinum; Bifidobacterium thermacidophilum* spp. *thermacidophilum; Bifidobacterium thermophilum; Bifidobacterium vansinderenii*).

**Figure 1:**
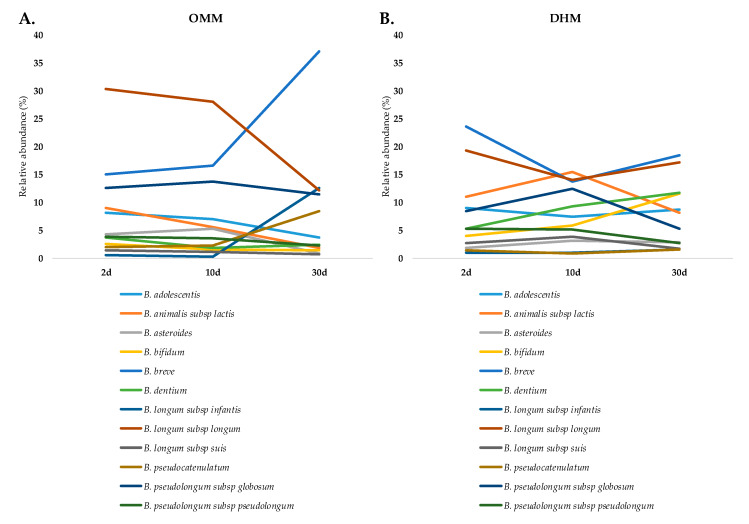
*Bifidobacterium* evolution. Evolution of *Bifidobacterium* species in (**A**) the own mother’s milk (OMM) group and (**B**) the donor human milk (DHM) group during the first month of life.

Premature babies fed with OMM showed *B. longum*, *B. breve,* and *B. pseudolongum* spp. *globosum* as their dominant species, which represented almost 75% of the bifidobacterial abundances during the first month of life. Interestingly, at one month of life, significant increases in the relative abundances of *B. breve* and *B. longum* spp. *infantis* were observed (Figure 1A). The three babies remaining in OMM at three months were dominated by seven species (Figure S2C; Table S2), mainly by *B. breve* and *B. longum* spp. *longum*, as corroborated by qPCR, and *B. pseudocatenulatum* (Figure S3A; Table S2). On the other hand, in the fecal microbiota of premature babies fed with donor milk, *B. breve* and *B. longum* ssp. *longum* were also the species with higher abundances at two days of life. However, other species, such as *B. bifidum*, *B. dentium*, *B. adolescentis,* and *B. animalis* spp. *lactis,* among others, increased over time (Figure 1B).

At two days, only *B. longum* spp. *suis* showed significantly (*p* < 0.05) higher abundances in the DHM than in the OMM group. However, the DHM led to significant increases in *B. animalis* spp. *lactis* (*p* < 0.01), *B. longum* spp. *suis* (*p* < 0.01), *B. bifidum* (*p* < 0.01), and *B. pseudolongum* spp. *pseudolongum* (*p* < 0.05) species at ten days, with respect to the OMM babies. At this age, *B. longum* spp. *longum*, *B. vansinderenii*, and *B. reuteri* presented significantly (*p* < 0.05) higher abundances in OMM babies. At one month of age, the diverse bifidobacterial microbiota in the DHM group also showed significantly higher abundances of *B. bifidum* (*p* < 0.01) and *B. dentium* (*p* < 0.05), which were also corroborated by qPCR (Table S2), and *B. animalis* spp. *lactis* (*p* < 0.01) and *B. magnum* (*p* < 0.05) relative to the OMM group.

Linear discriminant analysis effect size (LEfSe) was performed with the objective to determine the presence of any species that could discriminate between the two groups of babies based on feeding type during the sampling times analyzed. The DHM group was discriminated by several bifidobacterial species, with *B. thermacidophilum* spp. *thermacidophilum* having the greatest discriminatory power at two days of life, *B. animalis* spp. *lactis* and *B. bifidum* at ten days, and *B. biavatii*, *B. thermacidophilum* spp. *thermacidophilum,* and *B. bifidum* at one month of life, among others (Figure S4). In contrast, the OMM group of babies did not show any discriminatory species during the first days of life, likely due to the fact that all of them were dominated by a small number of species at that time. However, at three months of age, *B. pseudocatenulatum*, *B. longum* spp. *longum,* and *B. breve* showed discriminatory power in the OMM group (Figure S4).

### 3.3. Effect of Feeding on Gut Bifidobacteria Diversity

**Figure 2:**
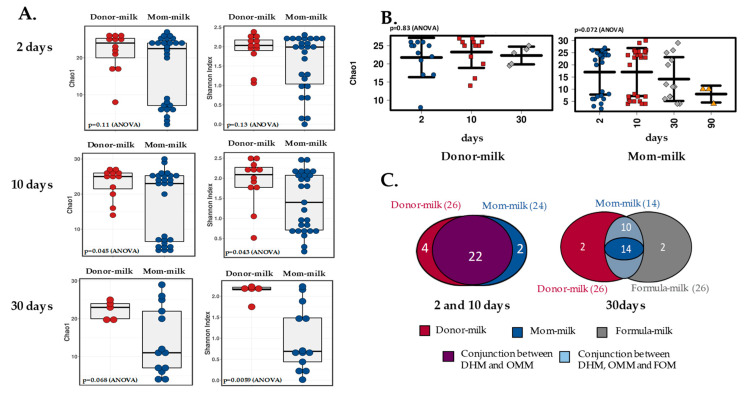
*Bifidobacterium* diversity. (**A**) Alpha diversity between groups. Chao1 and Shannon index comparisons between the DHM and OMM milk groups at 2 (*n* = 12 and *n* = 26, respectively), 10 (*n* = 12 and *n* = 27, respectively), and 30 (*n* = 5 and *n* = 13, respectively) days of life. (**B**) Alpha diversity over time. Chao1 index over time comparison in the DHM and OMM milk groups (*n* = 3 at 90 days). (**C**) Venn diagram. Number of unique and shared species in the babies’ groups over time. Colors and groups are explained in each figure.

When assessing alpha diversity in the premature feces, we observed that it differed between the OMM and DHM groups, even after several weeks, as indicated by different indexes (Chao 1 and Shannon). At two days of life, alpha diversity was similar between both infant groups, however, it became significantly higher in the DHM group at one month of life (*p* < 0.01, Shannon index) (Figure 2A). Moreover, this higher level of alpha diversity remained in the DHM group of babies when samples from formula-fed babies were incorporated in the comparison at 30 days of age (Figure S2A). Whereas feces from infants breastfed with OMM showed decreasing alpha diversity with increasing age, with lower levels at 90 days, babies fed with DHM exhibited almost constant diversity levels, with just slight changes at 30 days (Figure 2B). Consistent with these findings, the analysis of the unique and the shared species of the bifidobacterial community showed decreased species numbers in the OMM samples with age, while the DHM samples remained stable in regard to species numbers (Figure 2C). At three months, the Chao 1 index indicated lower diversity (*p* < 0.05) in the samples remaining in the breastfeeding group (OMM) with respect to the formula group, however, the Shannon index did not show any statistical differences (*p* = 0.85) (Figure S2B).

**Figure 3:**
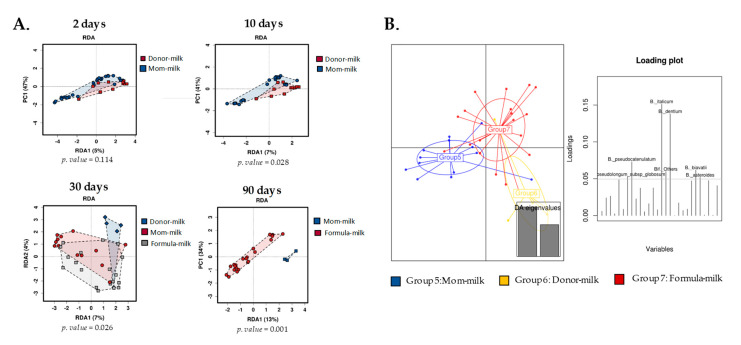
*Bifidobacterium* grouped based on feeding type. (**A**) Redundancy analysis (RDA) showing separation over time. (**B**) Discriminant analysis of principal components (DAPC) plot and loading plot at 30 days of life. Colors and groups are explained in each figure.

Using a multivariate redundant discriminant analysis (RDA), we also observed how the different feeding types at the beginning of life had significant impacts on the gut bifidobacterial structure. Statistically significant differences were observed between OMM and DHM groups during the first month of life (*p* < 0.05), and between the formula milk group and the remaining breastfeeding infants at 90 days (*p* < 0.01) (Figure 3A). Discriminant analysis of principal components (DAPC), which transforms data using principal component analysis (PCA) and subsequently identifies clusters using discriminant analysis (DA), showed that the OMM and DHM samples were clustered at the biggest distance at one month of age. This seemed to be mainly due to subtle changes in the abundances of species such as *B. dentium* and *B. pseudocatenulatum*, which indicated the presence of different microbial clusters (Figure 3B). The babies in the formula-feeding cluster were located in an intermediate position, as this group included babies from both the OMM and DHM feeding modes.

### 3.4. Feeding Impact on Microbiota Metabolism

**Figure 4:**
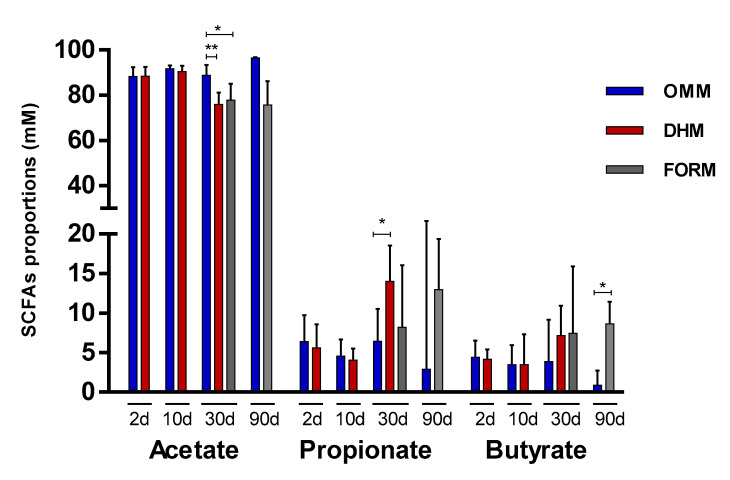
Proportions (% in mM) of the main short-chain fatty acids (SCFAs) determined by gas chromatography (GC) in the feeding type groups. Median and IQR range values are represented. OMM: own mother’s milk; DHM: donor human milk; FORM: formula milk group. * indicates a significant difference (* *p* < 0.05; ** *p* < 0.01).

Regarding SCFAs, acetate showed the highest levels in both the OMM (18.48, 24.64, 28.41, and 99.24 median mM levels at 2, 10, 30, and 90 days, respectively) and DHM (19.18, 28.73, and 22.29 median mM levels at 2, 10, and 30 days, respectively) groups. This was followed by propionate (1.11, 1.11, 1.85, and 4.35 median mM levels at 2, 10, 30, and 90 days, respectively, in the OMM group; 1.11, 1.16, and 6.16 median mM levels at 2, 10, and 30 days, respectively, in the DHM group). Butyrate showed the lowest concentrations (0.90, 0.90, 0.90, and 0.90 median mM at 2, 10, 30, and 90 days, respectively, in the OMM group; 0.90, 0.90, and 5.00 median mM levels at 2, 10, and 30 days, respectively, in the DHM group) (Table S3). Accordingly, the molar proportions of the SCFAs, which were calculated as the percentage of each acid with respect to the total (acetate + propionate + butyrate), showed acetate to be of the highest proportions in both the OMM and DHM groups, followed by propionate and then butyrate (Figure 4). No statistically significant differences were found during the first ten days of life according to the feeding mode. However, at one month of life, proportions of acetate were significantly higher in the OMM group with respect to the DHM (*p* < 0.01) and formula-fed (*p* < 0.05) babies. At this age, propionate exhibited lower proportions (*p* < 0.05) in the OMM group with respect to the DHM group. Higher proportions of acetate were also observed in the OMM feeding group compared with the formula group (a mix between previous OMM-fed and DHM-fed babies) at three months of life, although this did not reach statistical significance (*p* = 0.078). Remarkably, OMM feeding also led to significantly (*p* < 0.05) lower concentration of propionates (2.38–7.32 interquartile range (IQR)mM levels) with respect to the formula group (7.61–16.51 IQR mM levels) after 90 days of life (Table S3), as well as lower (*p* < 0.05) proportions of butyrate (Figure 4).

## 4. Discussion

Feeding mode is one of the most studied perinatal factors, with particular emphasis on the effects on the colonization and establishment of the infant gut microbiome during the early stages of life [1]. In recent years, there has been increasing encouragement directed toward breastfeeding practices both in term and preterm babies due to its beneficial properties and effects on homeostasis and health throughout the life course [29]. Breast milk is considered to be the main driver of the intestinal microbiota maturation [30], with a characteristic microbial profile observed in healthy breastfed babies [1,31]. One of the main microbial actors in the neonatal scenario is encompassed by bifidobacteria, which are among the first anaerobes to colonize the gut microbiota and constitute the dominant group in the intestinal microbiota of healthy full-term breastfed infants with respect to formula-fed or premature babies [1,3,31]. However, in premature neonates, correct gut microbial colonization is compromised due to various reasons, with *Bifidobacterium* being one of the most affected genera [3,4]. Despite the support and promotion of breastfeeding [11], this option is not always possible in preterm babies, with DHM being the preferred substitute and formula milk being the last option [11]. DHM is subject to a HoP that modifies some of its raw characteristics [8,13]; and it is known that premature babies fed with DHM show a gut microbiota profile different to that of OMM-fed babies [14,15,16].

To the best of our knowledge, this was the first study to assess the gut bifidobacterial composition at the species level in premature babies fed with human milk from donor mothers. Previous studies analyzed the effect of DHM on the overall gut microbiota composition of premature babies and observed lower abundances of the *Bifidobacterium* genus in the DHM group [14,15,16], however, the effect of this type of feeding at the species level was not previously explored. Our results demonstrated that the type of human milk fed to premature babies impacts their bifidobacterial population development. We found higher alpha diversity in bifidobacterial species in DHM-fed babies with respect to OMM-fed babies. These results, although interesting, are not surprising, since exclusive breast feeding was repeatedly associated with lower diversity in comparison with formula-feeding [31,32,33,34], favouring the development of a so-called “milk-oriented microbiota” with positive, long-lasting effects [1]. In the specific case of the bifidobacterial population and in opposition to some studies including the whole gut microbiota [14,15,16], OMM promoted lower diversity than DHM. These differences did not seem to be due to different early species levels, but rather to the evolution of the bifidobacterial microbiota, since at two days of life the alpha diversity was not different between the groups, but both diversity and the number of species detected decreased over time in the OMM group. At three months of life, the three babies still breastfeeding had a bifidobacterial profile dominated by seven main species. This profile demonstrated a clear separation of both groups into two different clusters according to both multivariate redundant discriminant analysis and discriminant analysis of principal components after one month of feeding, likely due to the face that, at birth, babies are colonized by a relatively large array of bifidobacteria from their mothers, which are later selected based on the baby’s feeding mode, i.e., OMM or DHM. In this setting, *Bifidobacterium* species able to metabolize the carbohydrates and HMOs present in breast milk would be able to increase in number and remain, however, those unable to metabolize HMOs would be dominated by the first. Several studies confirmed that carbohydrates are not affected by HoP process [8,13]; however, large variability was observed between different women, thereby suggesting a genetic background; therefore, milk carbohydrate composition may be an important driver of bifidobacterial populations [35,36]. In addition, the gut microbiota profile [14,15,16] may lead to differences in abundances of other HMO metabolizers, such as *Bacteroides* members, in the premature gut, which may interfere with the availability of carbon sources by bifidobacteria species through the process of crossfeeding [37].

The feces of OMM-fed premature babies showed *B. longum*, *B. breve,* and *B. pseudolongum* as the dominant species during the first month of life, and later *B. pseudocatenulatum.* We observed how, at one month of age, there was a shift in the subspecies of *B. longum*, with a decrement of *B. longum* ssp. *longum* and an increment of *B. longum* ssp. *infantis*. These findings were in concordance with the ability of some *Bifidobacterium* species to metabolize HMOs. In this way, *B. longum* ssp. *infantis* was the first bifidobacteria studied to carry a cluster of genes involved in the metabolism of HMOs [38], with *B. longum* ssp. *longum* [39] and *B. breve* [40] also able to utilize some types of HMOs. On the other hand, DHM-fed premature babies’ feces exhibited increments over time of different species, such as *B. bifidum*, *B. dentium*, *B. adolescentis*, and *B. animalis* spp. *lactis*, among others, corroborating the higher diversity of bifidobacterial members at one month of life in this group of babies.

Following the trend observed regarding bifidobaterial composition, no differences in the proportions of SCFAs between the DHM and OMM groups were found until one month of life. At this age, the OMM group exhibited significantly higher proportions of acetate, suggesting higher microbial metabolism of acetate producers, such as bifidobacteria, and better adaptation to this environment. Higher proportions of propionate were observed in the DHM group, possibly due to a lower capability of their bifidobacteria to produce acetate or changes in other propionate-producing gut members due to crossfeeding. These differences were maintained at three months in the three babies that continued to feed with their own mother’s milk.

It is important to point out that this study, although pioneering in terms of its field, had some limitations, mainly linked to the relatively small number of infants and the large interindividual variability, which are very common limitations in studies regarding preterm babies’ microbiota. Moreover, other confounding factors, such us the use of perinatal antibiotics which is strongly linked to prematurity, could also have affected these results. A larger follow-up would give us a better idea of the impact of DHM in the long-term. However, it is also true that this study focused on the reality at NCUs, where is difficult to provide DHM for more than one month; therefore, this study lays the basis for larger studies on the relationship between human bank milk and the development of bifidobacterial populations in early life.

## 5. Conclusions

This study highlighted the effect of feeding mode on gut microbiota establishment in premature babies at the bifidobacterial species level. Differences in specific *Bifidobacterium* species composition were shown between DHM-feeding and breastfeeding, which may contribute to the development of specific new strategies or treatments aimed at minimized the impact of this feeding type, which is the preferred option before formula milk at NCUs. Further studies are needed to unveil the long-term effects of DHM and to enhance the processing of human milk to preserve its key components.

## Figures and Tables

**Table 1:** Primers and annealing temperatures used in this study for *Bifidobacterium* species quantification by qPCR.

Target	Primer Sequence (5’-3’)	T (°C)	Reference
*Bifidobacterium bifidum*	TGACCGACCTGCCCCATGCT	61	[21]
	CCCATCCCACGCCGATAGAAT		
*Bifidobacterium breve*	AATGCCGGATGCTCCATCACAC	61	[21]
	GCCTTGCTCCCTAACAAAAGAGG		
*Bifidobacterium catenulatum* group	GCCGGATGCTCCGACTCCT	64	[21]
	ACCCGAAGGCTTGCTCCCGAT		
*Bifidobacterium longum* group	TTCCAGTTGATCGCATGGTCTTCT	65	[21]
	GGCTACCCGTCGAAGCCACG		
*Bifidobacterium dentium*	ATCCCGGGGGTTCGCCT	61	[22]
	GAAGGGCTTGCTCCCGA		
*Bifidobacterium adolescentis* group	CTCCAGTTGGATGCATGTC	61	[22]
	CGAAGGCTTGCTCCCAGT		
*Bifidobacterium angulatum*	CAGTCCATCGCATGGTGGT	61	[22]
	GAAGGCTTGCTCCCCAAC		
*Bifidobacterium animalis*	ACCAACCTGCCCTGTGCACCG	67	[23]
	CCATCACCCCGCCAACAAGCT		

## Supplementary Materials

Supplementary materials can be found at https://www.mdpi.com/2076-2607/8/5/760/s1.
