# Supplementary material for: Donated Human Milk as a Determinant Factor for the Gut Bifidobacterial Ecology in Premature Babies
Source: Microorganisms. 2020 May 19;8(5):760. doi: 10.3390/microorganisms8050760 (PMC7285294; doi:10.3390/microorganisms8050760)
Supplement: Supplementary file 1 [file microorganisms-08-00760-s001.pdf]

Supplementary Tables:

Table S1. Perinatal characteristics of premature babies. BW (birth weight), GA (gestational age), DH (days at hospital), AB (antibiotics). OMM: own mother milk; DHM: donor human milk; FORM: formula milk groups

		**BW (gr, mean±SD)**	**GA (weeks, mean±SD)**	**DH (days, mean±SD)**	**Delivery mode (% vaginal)**	**Gender (% girl)**	**Infant AB (%)**	**Mother AB (%)**
**2d**	**OMM** (n=26)	1351.73±353.31	30.88±2.44	46.04±26.98	46.15	38.46	80.77	3.85
	**DHM** (n=12)	1246.67±302.42	30.25±2.70	50.17±33.09	41.67	50.00	75.00	16.67
	p. value	*0.23*	*0.40*	*0.98*	*0.80*	*0.50*	*0.69*	*0.17*
**10d**	**OMM** (n=27)	1332.78±341.50	30.89±2.38	46.44±26.14	44.44	37.04	77.78	3.70
	**DHM** (n=12)	1240±296.55	29.92±2.43	50.83±32.61	41.67	58.33	75.00	16.67
	p. value	*0.30*	*0.15*	*0.99*	*0.87*	*0.22*	*0.85*	*0.16*
**30d**	**OMM** (n=13)	1273.46±362.51	30.62±3.28	51.31±31.01	53.85	38.46	84.62	7.69
	**DHM** (n=5)	1007±257.04	28.40±1.81	79±33.70	40.00	40.00	60.00	20.00
	**FORM** (n=20)	1409.75±279.91	31.43±1.36	39.25±17.94	35.00	45.00	80.00	5.00
	p. value	*0.04 (DHM vs FORM)*	*0.04 (DHM vs FORM)*	*0.03 (DHM vs FORM)*	*0.56*	*0.93*	*0.51*	*0.54*
**90d**	**OMM** (n=3)	1235±564.51	29.67±5.13	62.33±57.95	66.67	33.33	100.00	0.00
	**FORM** (n=23)	1287.17±357.78	30.55±1.97	50.22±27.8	34.78	47.83	78.26	4.35
	p. value	*1.00*	*1.00*	*1.00*	*0.29*	*0.64*	*0.37*	*0.71*

Table S2. Levels (Log n° cells /gram of feces) of different *Bifidobacterium* species determined by quantitative PCR in the feeding type groups. Median and interquartile range values are represented. OMM: own mother milk; DHM: donor human milk; FORM: formula milk groups; BLD: below detection limit. OMM 90 days: median (mín-max) showed. LD: 4.36 (*B. bifidum*), 5.37 (*B. breve*), 5.30 (*B. catenulatum*), 4.28 (*B. dentium*), 4.73 (*B. longum*), 5.14 (*B. angulatum*), 4.00 (*B. adolescentis*) 4.93 (*B. animalis*).

		B. bifidum	*B. breve*	*B. catenulatum*	*B. dentium*	*B. longum*	*B. angulatum*	*B. adolescentis*	*B. animalis*
**2 days**	**OMM**	BDL	BDL	BDL	BDL (BDL - 4,43)	4,74 (BDL - 4,94)	5,22 (5,17 - 5,26)	BDL	4,96 (BDL - 5,67)
	**DHM**	BDL (BDL - 4,58)	BDL	BDL	BDL	4,95 (BDL - 5,52)	5,17 (BDL - 5,21)	BDL	4,93 (BDL - 5,24)
**10 days**	**OMM**	4,44 (BDL - 4,63)	BDL	BDL	BDL	4,79 (BDL - 5,22)	5,19 (5,17 - 5,29)	BDL (BDL - 4,10)	5,08 (BDL - 5,64)
	**DHM**	BDL (BDL - 4,47)	BDL	BDL (BDL - 5,47)	BDL (BDL - 4,59)	BDL (BDL - 6,16)	5,17 (BDL - 5,23)	BDL	BDL (BDL - 5,53)
**30 days**	**OMM**	BDL (BDL - 4,80)	5,83 (BDL - 8,67)	BDL (BDL - 5,45)	BDL (BDL - 4,66)	6,11 (BDL - 8,49)	5,24 (BDL - 5,28)	BDL	5,32 (BDL - 5,87)
	**DHM**	4,52 (4,40 - 6,35)	BDL (BDL - 5,45)	BDL	4,52 (BDL - 7,47)	4,89 (BDL - 5,36)	5,20 (5,18 - 5,25)	BDL	5,20 (BDL - 5,42)
	**FORM**	4,54 (BDL - 5,06)	7,80 (BDL - 8,98)	BDL	BDL (BDL - 5,15)	5,28 (BDL - 7,20)	5,18 (BDL - 5,24)	BDL (BDL - 4,15)	5,66 (5,08 - 5,96)
**90 days**	**OMM**	4,42 (BDL - 8,52)	8,88 (BDL - 9,40)	5,56 (BDL - 10,56)	BDL	8,15 (5,17 - 9,15)	5,19 (BDL - 5,22)	BDL	5,59 (BDL - 5,92)
	**FORM**	4,65 (BDL - 6,75)	7,86 (6,41 - 8,61)	BDL	BDL (BDL - 4,63)	5,31 (4,78 - 7,97)	5,18 (5,15 - 5,29)	BDL (BDL - 4,27)	5,67 (5,16 - 6,12)

Table S3. Levels (mM) of the main SCFAs determined by GC in the feeding type groups. Median and interquartile range values are represented. OMM: own mother milk; DHM: donor human milk; FORM: formula milk groups. OMM 90 days: median (min-max) showed. * indicate significant differences (*p*.value<0.05) among feeding groups.

		Acetate	Propionate	Butyrate	Total SCFAs
**2 days**	**OMM**	18,48 (10,24 - 24,36)	1,11 (1,11 - 1,11)	0,90 (0,91 - 0,90)	20,49 (12,25 - 26,39)
	**DHM**	19,18 (15,01 - 24,6)	1,11 (1,11 - 1,11)	0,90 (0,90 - 0,90)	21,77 (17,11 - 26,61)
**10 days**	**OMM**	24,64 (20,59 - 33,19)	1,11 (1,11 - 1,18)	0,90 (0,90 - 0,90)	26,73 (22,60 - 37,31)
	**DHM**	28,73 (24,04 - 34,18)	1,16 (1,11 - 2,15)	0,90 (0,90 - 2,36)	32,09 (26,05 - 38,46)
**30 days**	**OMM**	28,41 (22,2 - 63,15)	1,85 (1,11 - 4,41)	0,90 (0,90 - 3,26)	30,42 (26,71 - 76,94)
	**DHM**	22,29 (14,62 - 51,37)	6,16 (3,35 - 9,37)	5,00 (0,90 - 10,38)	38,02 (20,77 - 66,94)
	**FORM**	35,45 (28,71 - 57,86)	3,66 (2,51 - 6,43)	4,22 (1,46 - 7,65)	46,31 (36,32 - 69,38)
**90 days**	**OMM**	99,24 (24,96 - 144,71)	4,35 (2,38 - 7,32)*	0,90 (0,90 - 0,90)*	102,53 (33,19 - 149,97)
	**FORM**	69,49 (46 - 95,12)	12,34 (7,61 - 16,51)	6,04 (1,8 - 12,31)	82,01 (65,88 - 122,43)

Supplementary Figures:

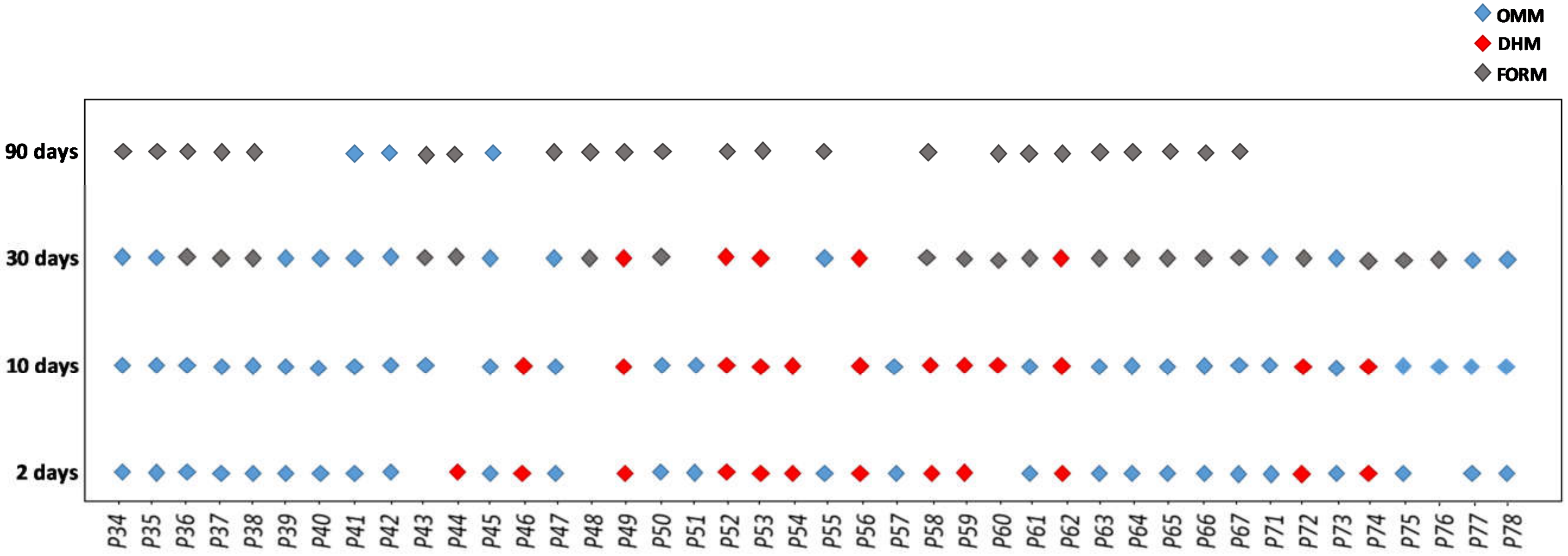

Figure S1. Feeding type distribution among the premature babies included in this study over the time. OMM: own mother milk; DHM: donor human milk; FORM: formula milk groups.

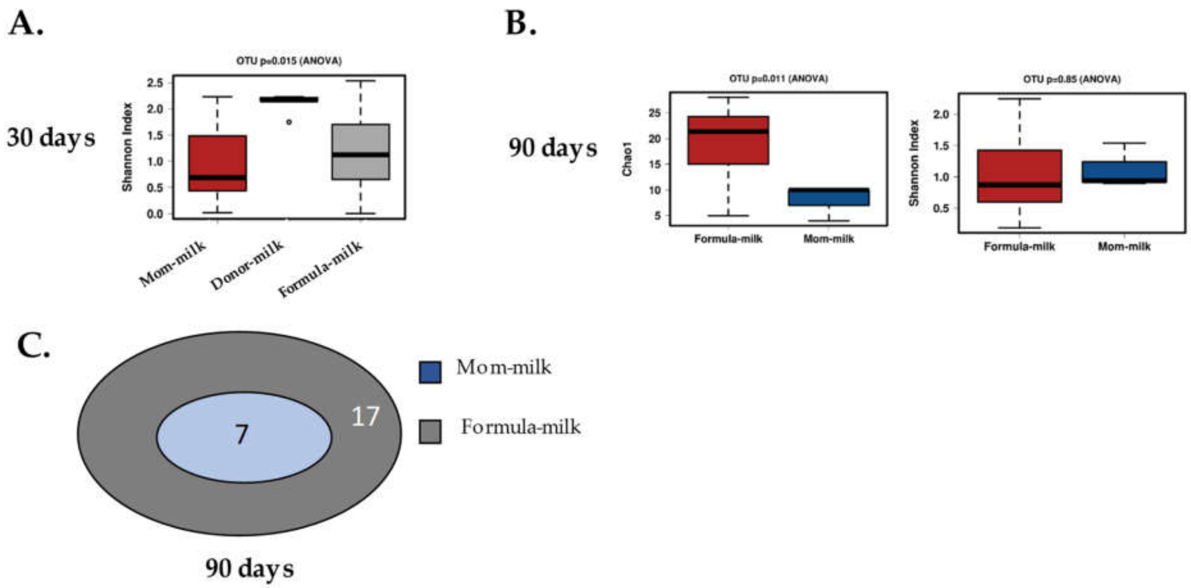

Figure S2. ***Bifidobacterium*** **diversity. (A)** Alpha-diversity between groups. Chao1 and Shannon indexes comparison between Donor (DHM), Mom (OMM) and Formula milk groups at 30 days of life. **(B)** Alpha-diversity over time. Chao1 and Shannon indexes comparison between Mom (OMM) and Formula milk groups at 90 days of life. **(C)** Venn diagram. Number of unique and shared species in the babies' groups at 90 days. Colours and groups explained in each figure.

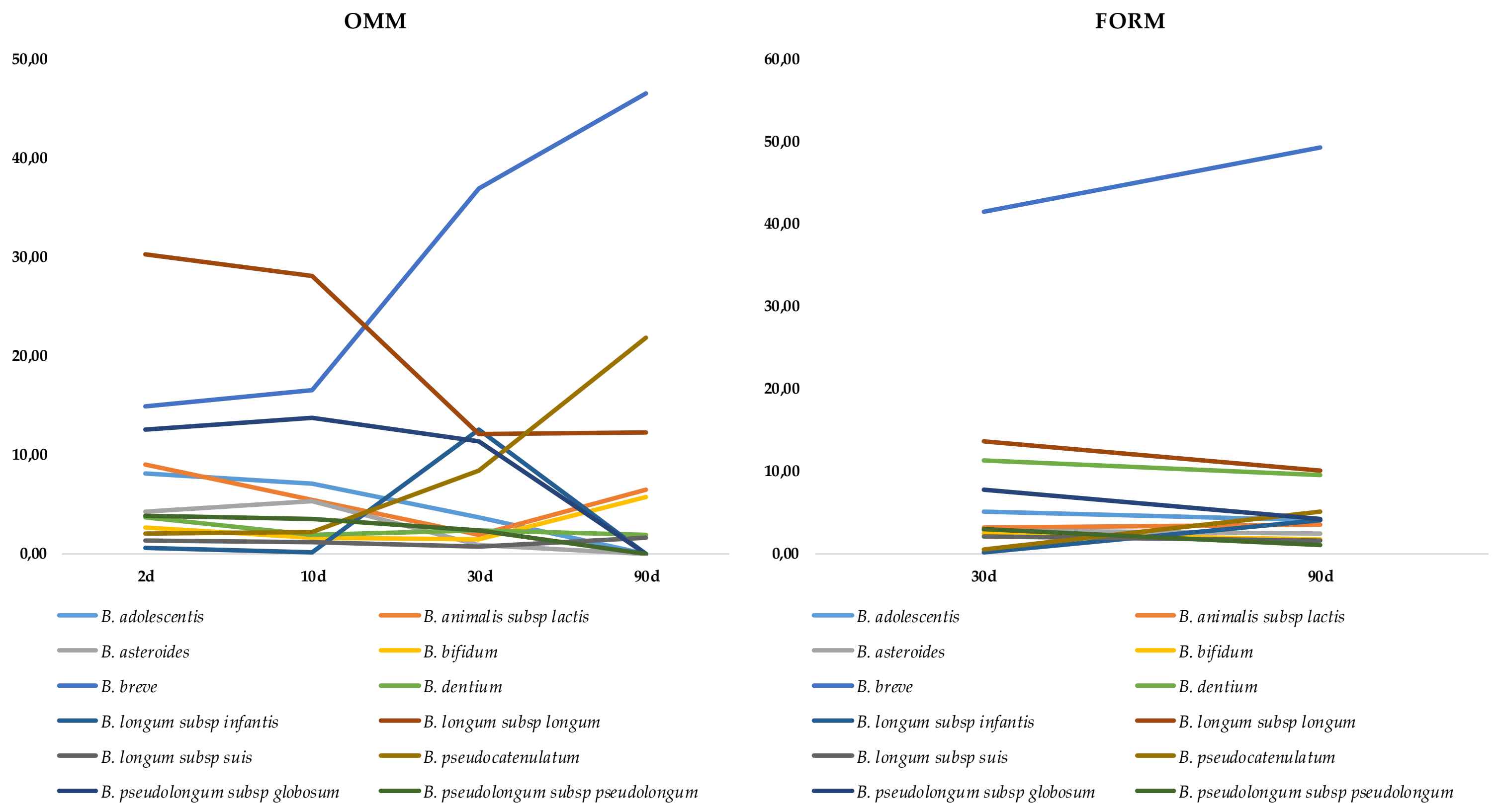

Figure S3. ***Bifidobacterium*** **evolution**. Evolution of *Bifidobacterium* species in **(A)** mom milk (OMM) group and in **(B)** formula milk (FORM) group during the first three months of life.

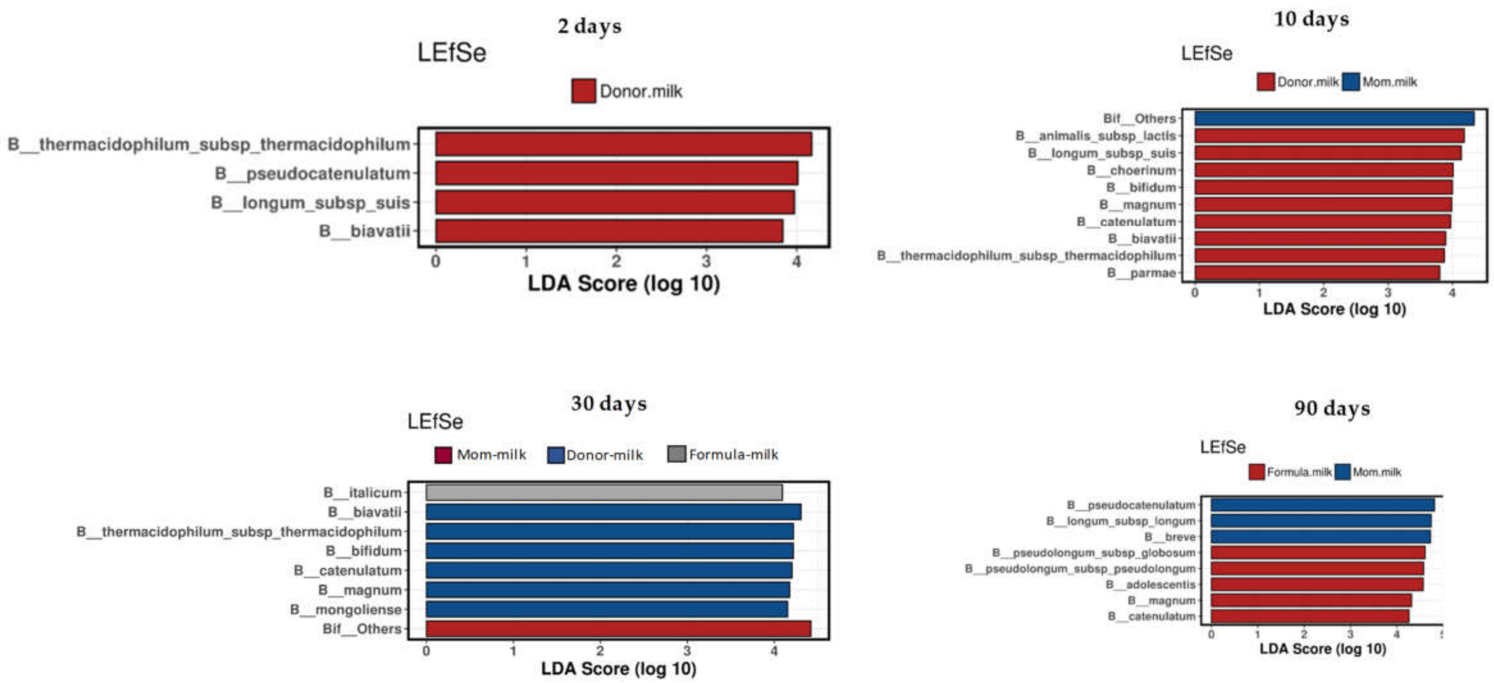

Figure S4. Differential species. Linear discriminant analysis (LDA) combined with effect size measurement (LEfSe) show the most differentially abundant *Bifidobacterium* species over time among feeding type. Colours and groups explained in each figure.
